# The clinical utility and costs of whole-genome sequencing to detect cancer susceptibility variants—a multi-site prospective cohort study

**DOI:** 10.1186/s13073-023-01223-1

**Published:** 2023-09-19

**Authors:** Aimee L. Davidson, Uwe Dressel, Sarah Norris, Daffodil M. Canson, Dylan M. Glubb, Cristina Fortuno, Georgina E. Hollway, Michael T. Parsons, Miranda E. Vidgen, Oliver Holmes, Lambros T. Koufariotis, Vanessa Lakis, Conrad Leonard, Scott Wood, Qinying Xu, Amy E. McCart Reed, Hilda A. Pickett, Mohammad K. Al-Shinnag, Rachel L. Austin, Jo Burke, Elisa J. Cops, Cassandra B. Nichols, Annabel Goodwin, Marion T. Harris, Megan J. Higgins, Emilia L. Ip, Catherine Kiraly-Borri, Chiyan Lau, Julia L. Mansour, Michael W. Millward, Melissa J. Monnik, Nicholas S. Pachter, Abiramy Ragunathan, Rachel D. Susman, Sharron L. Townshend, Alison H. Trainer, Simon L. Troth, Katherine M. Tucker, Mathew J. Wallis, Maie Walsh, Rachel A. Williams, Ingrid M. Winship, Felicity Newell, Emma Tudini, John V. Pearson, Nicola K. Poplawski, Helen G. Mar Fan, Paul A. James, Amanda B. Spurdle, Nicola Waddell, Robyn L. Ward

**Affiliations:** 1 QIMR Berghofer Medical Research Institute, 300 Herston Road, Herston QLD 4006, Brisbane, QLD Australia; 2 Faculty of Medicine, University of Queensland, Brisbane, QLD Australia; 3 Faculty of Medicine and Health, University of Sydney, L2.22 The Quadrangle (A14), Sydney, NSW 2006 Australia; 4 Australian Genomics, Melbourne, VIC Australia; 5 Centre for Clinical Research, University of Queensland, Brisbane, QLD Australia; 6 Children’s Medical Research Institute, University of Sydney, Westmead, NSW Australia; 7 Genetic Health Queensland, Royal Brisbane and Women’s Hospital, Herston, QLD Australia; 8 Tasmanian Clinical Genetics Service, Royal Hobart Hospital, Hobart, TAS Australia; 9 Parkville Familial Cancer Centre, Peter MacCallum Cancer Centre and Royal Melbourne Hospital, Melbourne, VIC Australia; 10 Genetic Services of Western Australia, King Edward Memorial Hospital, Subiaco, WA Australia; 11 Cancer Genetics Department, Royal Prince Alfred Hospital, Sydney, NSW Australia; 12 University of Sydney, Sydney, NSW Australia; 13 Monash Health Familial Cancer, Monash Health, Melbourne, VIC Australia; 14 Faculty of Medicine, Nursing and Health Sciences, Monash University, Melbourne, VIC Australia; 15 Cancer Genetics, Liverpool Hospital, Sydney, NSW Australia; 16 Department of Health, Genetic Services of WA, Subiaco, WA Australia; 17 Genomics, Pathology Queensland, Brisbane, QLD Australia; 18 Adult Genetics Unit, Royal Adelaide Hospital, Adelaide, SA Australia; 19 Faculty of Health and Medical Sciences, University of Western Australia, Perth, WA Australia; 20 Familial Cancer Services, The Crown Princess Mary Cancer Centre, Westmead Hospital, Westmead, NSW Australia; 21 Department of Medicine, University of Melbourne, Melbourne, VIC Australia; 22 Prince of Wales Clinical School, UNSW Medicine and Health, The University of New South Wales, Sydney, NSW Australia; 23 Hereditary Cancer Centre, Prince of Wales Hospital, Sydney, NSW Australia; 24 School of Medicine and Menzies Institute for Medical Research, University of Tasmania, Hobart, TAS Australia; 25 Genomic Medicine and Familial Cancer Clinic, Royal Melbourne Hospital, Melbourne, VIC Australia; 26 Adelaide Medical School, Faculty of Health and Medical Sciences, University of Adelaide, Adelaide, SA Australia; 27 Sir Peter MacCallum Department of Oncology, University of Melbourne, Melbourne, VIC Australia

**Keywords:** Familial cancer, Genetics, Variants, Whole-genome sequencing, Diagnostic testing, Health economics

## Abstract

**Background:**

Many families and individuals do not meet criteria for a known hereditary cancer syndrome but display unusual clusters of cancers. These families may carry pathogenic variants in cancer predisposition genes and be at higher risk for developing cancer.

**Methods:**

This multi-centre prospective study recruited 195 cancer-affected participants suspected to have a hereditary cancer syndrome for whom previous clinical targeted genetic testing was either not informative or not available. To identify pathogenic disease-causing variants explaining participant presentation, germline whole-genome sequencing (WGS) and a comprehensive cancer virtual gene panel analysis were undertaken.

**Results:**

Pathogenic variants consistent with the presenting cancer(s) were identified in 5.1% (10/195) of participants and pathogenic variants considered secondary findings with potential risk management implications were identified in another 9.7% (19/195) of participants. Health economic analysis estimated the marginal cost per case with an actionable variant was significantly lower for upfront WGS with virtual panel ($8744AUD) compared to standard testing followed by WGS ($24,894AUD). Financial analysis suggests that national adoption of diagnostic WGS testing would require a ninefold increase in government annual expenditure compared to conventional testing.

**Conclusions:**

These findings make a case for replacing conventional testing with WGS to deliver clinically important benefits for cancer patients and families. The uptake of such an approach will depend on the perspectives of different payers on affordability.

**Supplementary Information:**

The online version contains supplementary material available at 10.1186/s13073-023-01223-1.

## Background

Inherited predisposition to cancer, due to germline variation in cancer susceptibility genes, accounts for a small but significant proportion of cancer diagnoses [1, 2]. To date, over 100 ‘familial’ cancer predisposition genes have been identified and the number is growing [3]. Pathogenic variants in these genes confer an increased lifetime risk for developing disease [4]. Thus, the identification of such variants provides important opportunities for personalised clinical intervention in the presenting individual, and for preventive disease risk management in biologically related unaffected carriers [4].

Suspected familial cancer cases are referred to specialist genetics facilities; in Australia these include Familial Cancer Centres (FCCs). Clinical genetic testing is conducted conventionally in a phenotype-directed manner, and to date has generally been limited to identifying disease-causing pathogenic variants in a single gene or small gene panel. WGS presents a phenotype-agnostic testing approach for the diagnosis of genetic conditions and, importantly, can be used for research studies. As WGS provides the most comprehensive genomic profile and includes regions and forms of pathogenic variants typically not covered by other genetic testing methods (for example non-coding regions or copy-number variants), it may be coupled with a range of different analyses without the need for multiple genetic tests [5, 6]. The identification of disease-causing pathogenic variants, for example, can be limited to a virtual panel of genes (equivalent to traditional phenotype-directed testing) or to coding regions only (equivalent to whole-exome testing). Additionally, as the clinical context of the tested individuals changes over time or as gene-phenotype relationships are established or evolve, WGS data can be reanalysed to lead to a new diagnosis [7, 8]. However, there are no studies which provide an accurate assessment of the clinical utility and economic feasibility of implementing WGS as a diagnostic tool in a familial cancer setting. Therefore, this study used WGS coupled with a virtual gene panel analysis to identify actionable, diagnostic disease-causing variants. Specifically, we compared the overall diagnostic yield and likely costs of this comprehensive WGS-based testing method to current standard of care for individuals with a suspected diagnosis of hereditary cancer. The study participants were recruited through the Inherited Cancer Connect Partnership (ICCon), a national collaboration of clinicians and scientists that encompasses the major FCCs around Australia [9].

## Methods

### Study cohort

Candidate study participants with a personal history of tumour-associated phenotype(s) were identified through 11 Australian FCCs (located within Queensland, New South Wales, Victoria, South Australia, Tasmania and Western Australia) between 2017 and 2020 as part of their standard clinical care. For the purposes of this study (hereafter referred to as the ICCon study), a tumour-associated phenotype was inclusive of both primary cancers and cancer gene syndrome-associated benign tumours. Specific inclusion criteria applied for this study are detailed in Table 1; however, if the individual met these inclusion criteria solely on the basis of a family history of: breast cancer, ovarian cancer (epithelial), colorectal cancer (in absence of unexplained polyposis), melanoma (in populations with high occurrence of melanoma), or prostate cancer, they were deemed ineligible. Eligibility was determined by the FCC clinicians and/or genetic counsellors, with additional consultation by the ICCon study leads if required. A total of 195 individuals (hereafter referred to as index cases) accepted enrolment into the study (Additional file 1: Table S1). The tumour-associated phenotype diagnoses for index cases were categorised and recorded into divisions as per Table 2. Tumour-associated phenotypes in the same tissue type and organ were considered separate primary diagnoses if the medical record denoted them as distinct.

**Table 1:** Eligibility criteria for recruitment of index cases

Eligibility criteria	Index cases (number / percent)^d^
An index case in a family that fulfils clinical criteria for a hereditary cancer syndrome	28 (14.4)
An index case with two primary cancers (either synchronous or metachronous) diagnosed at 60 years or younger	83 (42.6)
An index case with three primary cancers diagnosed at 70 years or younger	52 (26.7)
Index case with colorectal polyposis defined as per InSiGHT^a^ or eviQ^b^ testing guidelines for *MUTYH* and/or *APC*	29 (14.9)
A cancer-affected individual fulfilling two or more of the following criteria: • Early age at onset (under 40 years for adult cancer or < 10 years younger than the mean age of cancer diagnosis for that tumour type and sex^c^) • One or more first-degree relatives with the same kind of cancer • Two or more first- or second-degree relatives with different cancers, where at least one cancer is very rare	42 (21.5)

^a^The International Society for Gastrointestinal Hereditary Tumours (InSiGHT), as per https://www.insight-group.org/

^b^eviQ, as per https://www.eviq.org.au/

^c^Australian Institute of Health and Welfare 2017. Cancer in Australia 2017. Cancer series no. 101. Cat. no. CAN 100. Canberra: AIHW

^d^Percent does not add to 100% as index cases may have met more than one eligibility criteria

**Table 2:** Characteristics of the recruited index cases

Demographic	Index cases	
	Number	Percentage
Sex		
Female	141	72.3
Male	54	27.7
Age at first diagnosis		
18 and under	16	8.2
19–49	125	64.1
50 and over	54	27.7
Prior genetic testing		
Uninformative result^a^	140	71.8
None	55	28.2
Recruitment site		
QLD	46	23.6
VIC	58	29.7
TAS	14	7.2
SA	21	10.8
NSW	29	14.9
WA	27	13.8
Study referral		
Meets direct eligibility requirements	157	80.5
Eligible via independent panel assessment	38	19.5
Virtual panel used		
Panel A (107 genes)	24	12.3
Panel B (101 genes)	171	87.7
Broad tumour-associated phenotype^b^		
Gynaecological and breast		
Breast	43	22.1
Endometrial	12	6.2
Ovarian	9	4.6
Cervical	4	2.1
Other gynaecological	2	1.0
Urogenital		
Renal cell carcinoma	35	17.9
Prostate	6	3.1
Urothelial carcinoma	3	1.5
Other urogenital	13	6.7
Neuroendocrine		
Thyroid	29	14.9
Non-thyroid	26	13.3
Polyposis		
Adenomas or adenomatous polyposis	28	14.4
Serrated polyps	6	3.1
Hyperplastic polyps	6	3.1
Hamartomatous or Juvenile polyps	1	0.5
Other polyps	6	3.1
Gastrointestinal		
Colorectal	28	14.4
Upper gastrointestinal	11	5.6
Skin cancer		
Melanoma	21	10.8
Non-melanoma	14	7.2
Sarcoma		
Soft tissue	10	5.1
Bone	4	2.1
Other sarcoma	3	1.5
Respiratory	12	6.2
Head and neck	8	4.1
Brain	8	4.1
Lymphoma	8	4.1
Non-endocrine pancreatic	7	3.6
Unknown primary	3	1.5
Non-brain neurological	1	0.5
Other	43	22.1

^a^Inclusive of immunohistochemistry (IHC) analysis of somatic tissue and other somatic tissue testing in addition to germline genetic testing

^b^Percentage may not add to 100% as index cases may have been diagnosed with more than one tumour-associated phenotype

For each index case, there was either a clinical suspicion of a cancer predisposition syndrome, but there were no recommended tests for that specific presentation, or previous routine genetic testing had not identified a germline molecular genetic diagnosis for their presenting tumour-associated phenotypes. A further 19 family members of 12 index cases were also recruited to aid in the clinical interpretation of germline variants identified in the index cases. Study data were collected and managed using REDCap electronic data capture tools [10, 11]. Changes in risk management strategies were recorded in REDCap [10, 11] as per Additional File 1: Table S2. Written informed consent was provided by all participants, or by the relevant next of kin where the index case was under 18 years or deceased at time of recruitment.

### Virtual gene panel

Our analysis was restricted to a comprehensive virtual panel of cancer predisposition genes. The initial panel (panel A) comprising 107 genes was compiled by the ICCon study working group members from a larger panel described previously [12]. During the ICCon study, a revised panel of 101 genes was adopted (panel B). This revision of panel A was triggered following: review at the *Familial Aspects of Cancer* conference in 2018; consideration of the study conducted by Tudini et al. [12]; and acknowledgement of suggestions made during previously conducted multidisciplinary team (MDT) meetings. Panel A was used for the analysis of the first 24 participants, whilst panel B was used for the remaining 171 participants (Additional File 1: Table S3 and S4).

### Whole-genome sequencing

Germline DNA was extracted from peripheral blood, obtained as per the standard care practices of the participant’s respective FCC. DNA (1.2 to 10 µg) was supplied to The Kinghorn Centre for Clinical Genomics at the Garvan Institute of Medical Research (Sydney, Australia). Sequence libraries were generated from 500 ng DNA using the KAPA Hyper PCRFree Library Preparation kit and sequenced using 150 bp paired-end sequencing reads on the Illumina HiSeq X Ten platform (Illumina, San Diego, CA, USA) to a targeted read depth of 30X. FASTQ files were processed and analysed at the QIMR Berghofer Medical Research Institute. Sequence reads were trimmed using Cutadapt version 1.9 [13] and aligned to the GRCh37 reference genome using BWA-MEM from BWA Kit version 0.7.12 [14]. Duplicate alignments were marked with Picard (version 1.129, http://picard.sourceforge.net), and BAM files were coordinate-sorted using Samtools version 1.8 [15]. Mean read depth across each participant’s germline genome was determined using NGSCheck (genomiQa Pty. Ltd.). Mean read depth per genome was 34.3X (range 27.8X to 58.3X).

### Germline variant identification and prioritisation

Analysis was conducted in an ongoing manner as participants were recruited and sequenced. Single-nucleotide variants (SNVs) were identified using a dual calling strategy using GATK HaplotypeCaller [16] and qSNP [17]. Small insertions and deletions (indels) were identified using the GATK HaplotypeCaller. See Github public code repository under the AdamaJava project (https://github.com/AdamaJava) for details regarding software packages. SNVs and indels were annotated using the Ensembl Variant Effect Predictor (VEP) (v92.3) [18] for gene loci association and other bioinformatics predictions, with publicly available and in-house plugins. Unless present in ClinVar [19] with a likely pathogenic or pathogenic classification (as per the Clinvar May 2018 XML release), variants were disregarded as follows: variants with a minor allele frequency (AF) of > 1% in any of the major subpopulations (*n* ≥ 2000 individuals) of gnomAD (v2.1.1) [20], considering exome-derived and genome-derived allele frequencies separately; > 1% frequency within the ICCon study cohort; or > 1% in-house germline whole-genome dataset (*n* = 312). SNVs and indels with a variant allele fraction of less than 30 or 20% respectively were removed from further analysis.

Structural variants (SV, inversions, insertions and intra/inter-chromosomal translocations) and copy-number variants (CNV, deletions and duplications) were detected using DELLY (downloaded 20180903) [21] with default parameters (as per the recommended germline SV calling pipeline). CNVs were detected using CNVnator (downloaded 20190820) [22], with a bin size of 100 and the ‘ngc’ option given. CNVs called by CNVnator with a q0 value of < 0 or > 0.5 (unless the genotype score was < 0.25) were flagged for removal. CNVs called by only a single tool were flagged for manual review. Overlap between tools was defined as the same CNV type with ≥ 80% reciprocal overlap. SVs and CNVs that overlapped (≥ 80% for deletions, duplications and inversion; or any overlap for translocations and insertions) with exclusionary genome regions (consisting of the ENCODE Duke and DAC poor mappability regions [23], repetitive and low complexity regions [24], GRCh37 ‘N’ repeat regions, and regions where mapping quality frequently falls below MAPQ of 10) were flagged for exclusion. Likewise, variants with a > 5% AF within any of the gnomAD SV dataset (version 2.1) [25], in-house germline dataset or within-cohort were also flagged for exclusion. The in-house germline dataset frequencies were calculated from variants called using the same methods used for the ICCon study cohort. Overlap for frequency assignment was ≥ 80% for deletions, duplications and inversions, whilst the frequencies of variants within a 100-bp window were considered for translocations and insertions. Overlaps were determined using BEDTools versions 2.27.1 and 2.29.0 [26]. SV and CNV events were associated with genes via annotation with ENSEMBL (version 75) protein-coding gene footprint region (defined as 2000 bp upstream of the transcription start position to the transcription end position). Any degree of overlap between a CNV and gene locus was considered. For other SVs, overlap of one or both breakpoints with a gene locus was considered.

### Variant classification and reporting

All variants initially prioritised as described above were manually reviewed using the Integrative Genome Viewer (IGV) (version 2.3.97) [27]. These variants were then curated using ACMG/AMP guidelines [28]. Variants classified as being of uncertain significance (VUS), likely pathogenic (LP) or pathogenic (P) were reported in the form of a preliminary research report to an MDT meeting for clinical evaluation (Additional File 1: Table S4). Variants that did not meet strict ACMG/AMP criteria for benign (B) or likely benign (LB) classification were not included in the preliminary research reports for MDT meeting evaluation if they were as follows: a missense variant with a REVEL [29] score less than 0.5; a synonymous variant with no predicted impact on splicing; or an in-frame indel with a PROVEAN [30] score greater than − 2.5. However, these exclusionary conditions were not applied to the variant: if there was an apparent gene-phenotype association; an alternative bioinformatics predictor (such as CADD > 20 [31]) suggesting the variant to be deleterious; and/or the variant was present in ClinVar (at the time of manual review during curation) with a LP/P classification from at least one submitter. All variants classified as LP/P, even if for recessive conditions and/or conditions that did not clearly associate with the index cases’ presenting phenotype/s were considered for MDT meeting review. The MDT meeting panel was comprised of the following: the project coordinator; two or more variant curation and/or genomics researchers; and the treating clinician(s) and/or genetic counsellor(s). Variants were critically reviewed within the context of available clinical data, family history, bioinformatic and literature-derived evidence available at the time of clinical review until a consensus regarding the variant’s pathogenicity was reached. In some cases, supporting evidence was not sufficient to clearly classify the variant and further laboratory testing and/or expertise to assist with the interpretation, was sought to reach a consensus in the classification. Once a consensus was reached, a post-MDT meeting research report, summarising reportable variants, was issued to the treating clinician. For post-MDT meeting classification, VUS were further classified into three tiers following a system similar to that adopted by the Victorian Clinical Genetics Services [7, 32]. Namely, VUS:A—available evidence is highly suggestive that the variant is disease-causing; VUS:B—available evidence is insufficient to classify the variant as either disease-causing or benign; and VUS:C—available evidence is highly suggestive that the variant is likely benign. Variants downgraded to B/LB during the MDT meeting were not returned to the treating clinician (Additional File 1: Table S4). A pathogenic variant identified in two participants, I129 and I134, was returned without formal MDT meeting review following consultation with their Familial Cancer Clinic and lead study team. Clinical confirmation of variants reported post-MDT meeting was conducted by a NATA-accredited laboratory where the treating clinician wished to consider implementation of changes to risk management strategies.

### Health economic analysis

**Fig. 1:**
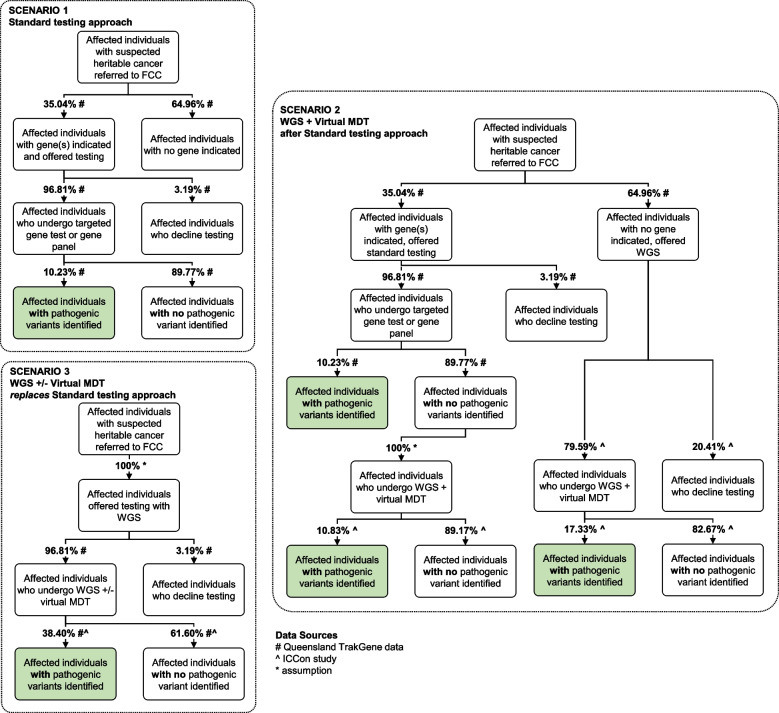
Scenarios used as the basis of the health economic analysis. Scenario 1: Standard testing; Scenario 2: standard testing followed by whole-genome sequencing (WGS); Scenario 3 upfront WGS. FCC—familial cancer centre; MDT—multi-disciplinary team meeting

We undertook an analysis to compare the costs and key outcome (overall diagnostic yield) associated with three hypothetical testing scenarios (Fig. 1). Scenario 1 describes a typical current approach to testing where affected individuals referred to FCCs are offered targeted gene or gene panel testing when specific gene(s) are indicated. Scenario 2 describes an approach where the option of WGS plus a virtual MDT meeting is offered to affected individuals in whom no gene(s) are initially indicated by an FCC, and to affected individuals in whom gene(s) were initially indicated but targeted gene or gene panel testing identified no LP/P variants. Scenario 3 describes an approach where all affected individuals referred to FCCs are offered WGS: where non-complex cases are offered WGS with follow-up by an FCC, and complex cases are offered WGS plus a virtual MDT meeting. A scenario using an agnostic panel testing approach for all cases was not assessed.

As our approach to the economic analysis is ‘programmatic’ (meaning we estimated the total likely cost of implementing each testing scenario as a national programme, taking into account ‘overheads’ to coordinate the programme as well as the resources required to conduct each test, including staff time and sequencing costs, Additional File 1: Table S5), we took a national, public health system perspective. All monetary values are expressed in Australian dollars. All resource utilisation estimates were costed using 2020 prices and pay rates, and inflation or deflation of costs was not applied. As all costs and benefits for a case were defined as accruing within the same 12-month period, no discounting was applied.

### Estimation of costs

The estimated costs included in the analysis account for staff time within FCCs to provide counselling before and after testing (where it is assumed that post-test costs are higher for individuals with positive test results, Additional File 1: Table S5), targeted gene and gene panel costs (expressed as an average cost per tested individual based on the relative distribution of cancer presentations to FCCs), WGS costs (based on data from the ICCon study), virtual MDT meeting costs (based on utilisation data from the ICCon study) and other costs associated with administering the WGS plus virtual MDT meeting testing approach for complex versus non-complex cases (Additional File 1: Table S5).

When identifying relevant costs from the ICCon study, we have been careful to exclude resource use that is specifically associated with a research setting (for example, ethics approvals and reporting), and to include resource use that would be expected to occur in clinical practice, outside of a research setting. The latter costs include the steps involved in genomic sequencing (that is, obtaining the appropriate biological sample(s), DNA extraction, library preparation, sequencing, analysis, data storage, and preparation of reports for clinicians [33]), clinician time to discuss and interpret the reports (whether that is time within or outside an MDT meeting), and programme co-ordination (such as, communication with FCCs, and co-ordination of virtual expert panel members).

The units of resource use observed in ICCon were averaged per genome, and then unit costs were applied. For the labour items, the monetary values used represent true costs to the health system, whereas the non-labour costs associated with genomic sequencing represent the prices charged by providers. Labour costs have been estimated by applying relevant pay rates from the University of Queensland and Queensland Health, noting that these are representative of pay scales in other Australian States and Territories. Capital costs associated with genomic sequencing are not included as a separate line item as it is assumed these would be captured in the prices charged by the providers.

### Uptake and diagnostic yield of testing scenarios

The uptake and diagnostic yield of testing applied in each scenario are shown in Fig. 1 and the total costs associated with each scenario are shown in Additional File 1: Tables S6 and S7. The proportion of affected individuals who are offered targeted gene or gene panel testing, and the proportion of those who accept this testing, were taken from State-wide de-identified and aggregated data from the FCCs in Queensland (provided by Queensland Health directly to the research team). The diagnostic yield for targeted gene or gene panel testing was taken from the same source.

The proportions of affected individuals who are offered and who undergo WGS in Scenario 2 were taken from the ICCon study. Separate proportions have been derived for (a) affected individuals who initially had gene(s) indicated and underwent targeted gene or gene panel testing with no LP/P variants identified, and (b) affected individuals who initially had no gene(s) indicated and had not been offered targeted gene or gene panel testing. The diagnostic yield for each of these patient subgroups ((a) and (b)) was also derived from the ICCon study. For affected individuals who would undergo upfront WGS (Scenario 3) assumptions have been made regarding the rate of uptake and the diagnostic yield, informed by results from the Queensland TrakGene data study and the literature.

The total number of affected individuals referred to all FCCs in Queensland in a year was available, but corresponding data for other States and Territories was not available. Consequently, the total number of affected individuals referred to all FCCs in Australia in a year has been estimated by scaling up the data from Queensland, using the populations from each jurisdiction.

### Sensitivity analyses

One-way sensitivity analyses were undertaken for selected parameters (Additional File 1: Table S6): applying a lower cost for WGS in Scenarios 2 and 3 ($1366 instead of $1750; based on a recent quote from a sequencing provider for a similar service); assuming all cases offered WGS in Scenario 3 will require a virtual MDT meeting (virtual MDT meetings only applied to complex cases in the base case); applying a higher rate of uptake of WGS in Scenarios 2 (96.8% instead of 79.6%); applying lower diagnostic yields for upfront WGS in Scenario 3 (17.3% or 28.2%, versus 38.4% in the base case); and assuming a lower and higher number of affected individuals are referred to an FCC, across all scenarios (50% and 150% of base case).

## Results

### Characteristics of the index cases

**Fig. 2:**
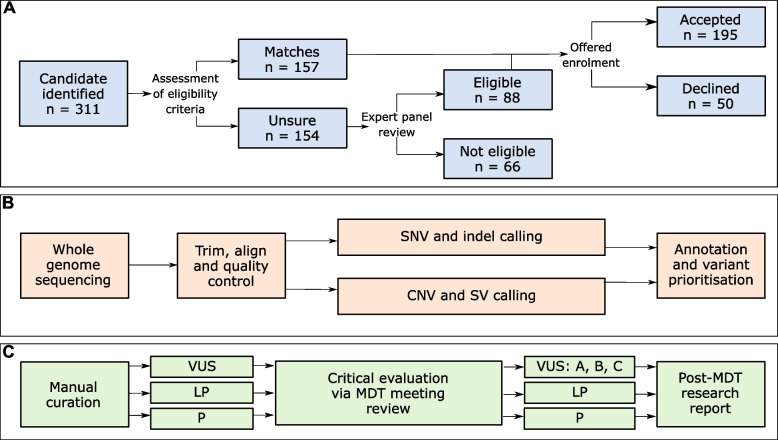
Schema of participant recruitment and approach to bioinformatics and curation.** A** Index case recruitment and enrolment acceptance. **B** Bioinformatics and variant identification approach. **C** Curation of prioritised variants. For detailed description of each step, see ‘Methods’. CNV—copy-number variant; indel—small insertions and deletions; LP—likely pathogenic; MDT—multidisciplinary team; P—pathogenic; SNV—single-nucleotide variant; SV—structural variant; VUS—variant of uncertain significance

Of the 311 study candidates identified at participating FCCs, 66 were deemed ineligible by expert panel review and a further 50 declined enrolment (Fig. 2). The 195 enrolled index cases were diagnosed with one to five tumour-associated phenotype(s) (median—2), with 72.3% diagnosed with their first phenotype before 50 years of age. Tumour-associated phenotype diagnoses were grouped into 15 overall categories, with > 15% of the index cases diagnosed with one or more gynaecological, urogenital, neuroendocrine, polyposis, gastrointestinal or skin associated phenotypes (Table 2, Additional File 1: Table S1).

### Germline variants of potential clinical significance identified within the index cases

**Fig. 3:**
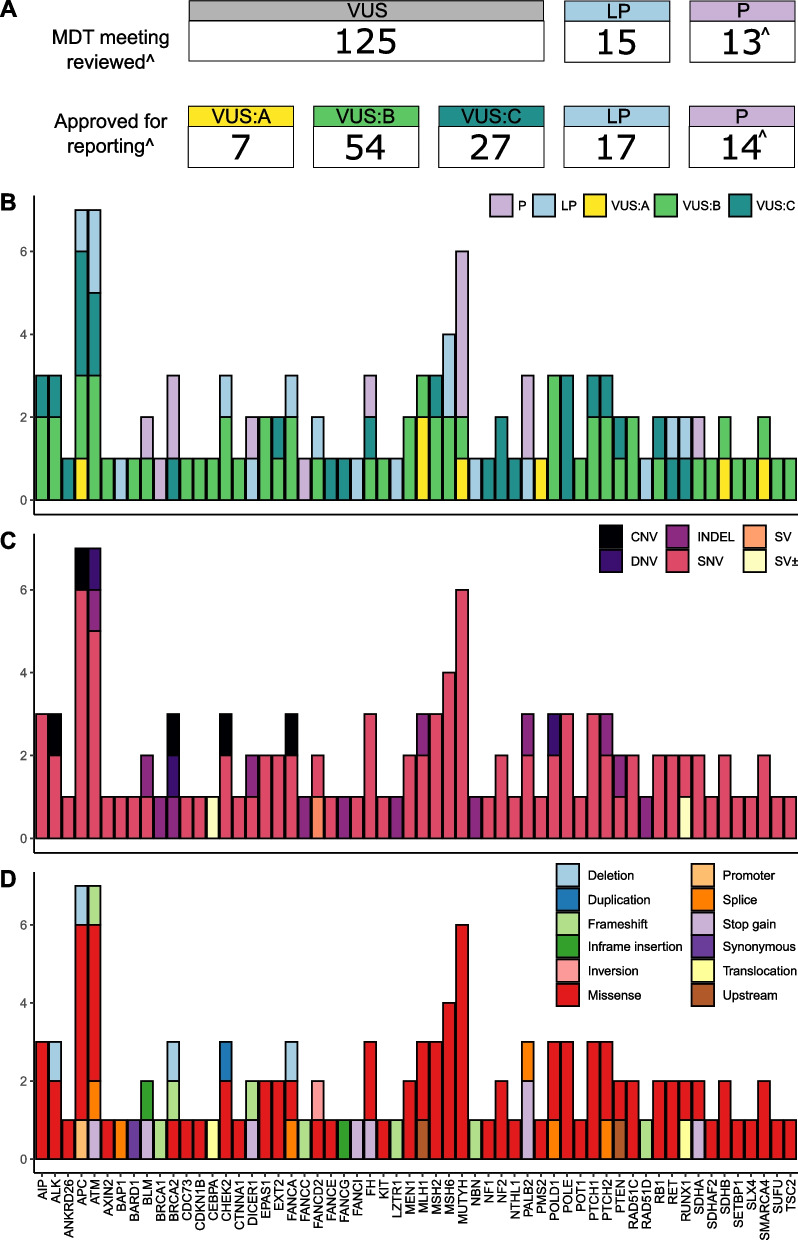
Variants identified within index cases.** A** The number and classification of variants discussed at the MDT meetings and returned to the treating clinician after the MDT meeting. Classifications: LP—likely pathogenic; P—pathogenic; VUS—variant of uncertain significance; VUS:A—available evidence is highly suggestive that the VUS variant is disease-causing; VUS:B—available evidence is insufficient to classify the VUS variant as either disease-causing or benign; and VUS:C—available evidence is highly suggestive that the VUS variant is likely benign. ^Two pathogenic *PALB2* variants known to be common within the Australian population were reported directly to the relevant Familial Cancer Centre without a formal MDT meeting. **B** Post-MDT meeting classification of returned variants. **C** Type of returned variants. Types: CNV—copy-number variant; DNV—dinucleotide substitution; INDEL—small insertion or deletion; SNV—single-nucleotide variant; SV—structural variant; SV ± —structural variant with somatic origin. **D** Molecular consequence of returned variants. For panels **B** through **D** the number and type of variants (*y*-axis) is shown for each gene (*x*-axis)

In total, 119 variants relating to 92 participants were returned to the treating clinician following MDT meeting evaluation. This included 31 LP/P variants and 88 VUS. (Fig. 3A, B, Table 3, Additional File 1: Table S4). Most VUS were sub-classified as VUS:B (54/88) with fewer sub-classified as the more clinically suspicious VUS:A (7/88) or less clinically suspicious VUS:C (27/88). Of the 31 LP/P variants, 10 were deemed as the causative germline variant for the presenting phenotype(s) of the index case. It is notable that of these 10 variants, seven would not have met current Australian clinical testing criteria for that gene (based upon eviQ guidelines reviewed August 2022) [34]. The remaining 21 variants were associated with new genetic diagnoses; providing a partial or incomplete genetic diagnosis; conferring carrier status for a recessive condition; or confirmed as being of somatic origin (Additional File 1: Table S4 and S8). The majority of participants received a post-MDT meeting report documenting a finding describing only a single variant (67/92). The remaining participants received findings for two (23/92) or three variants (2/92). Index cases (I148 and I176) received findings for two LP/P variants (Additional File 1: Table S4).

**Table 3:** Likely pathogenic and pathogenic variants considered for clinical actionability in this study

Gene	Classification	Variant (GRCh37)	Index case ID and presenting tumour-associated phenotype(s)
*APC*	LP	NM_000038.5:c.-38818_-27797del	I102: PS-A
*ATM*	LP	NM_000051.3:c.1402_1403del	I104: HL, NET-thyroid, URO-PC (metastatic)
		NM_000051.3:c.5644C>T	I189: NET-thyroid, URO-RCC
*BAP1*	LP	NM_004656.3:c.932-151G>A	I099: Brain, BAPoma
*BLM*	P	NM_000057.3:c.2695C>T	I121: URO-RCC, tunica albuginea cyst in scrotum
*BRCA1*	P	NM_007294.3:c.68_69del	I142: NET-thyroid, ALL
*BRCA2*	P	NM_000059.3:c.7805+473_8754+726del	I031: BC, CRC
		NM_000059.3:c.3847_3848del	I059: Cholangiocarcinoma
*CHEK2*	LP	NM_007194.3:c.349A>G	I090: Head and neck, NET-thyroid (papillary)
*DICER1*	LP	NM_177438.2:c.3300dup	I167: PS-H
	P	NM_177438.2:c.2556T>A	I058: OC (Sertoli Leydig); NET-thyroid (follicular)
*FANCA*	LP	NM_000135.2:c.1007-1G>A	I150: BC, EC
*FANCC*	P	NM_000136.2:c.67del	I148: Upper GI, URO (mediastinal germ cell tumour)
*FANCD2*	LP	NM_033084.3:c.783+385_1134+625inv	I114: OC, sarcoma (soft)
*FANCI*	LP	NM_001113378.1:c.511C>T	I056: OC (benign steroid cell tumour), bilateral benign serous cystadenofibromas, NET (lung carcinoid)
*FH*	P	NM_000143.3:c.1052C>A	I046: Leiomyosarcomas
*LZTR1*	LP	NM_006767.3:c.502del	I191: Cervical, melanoma, NET-thyroid, respiratory, history of other benign tumours
*MSH6*	LP	NM_000179.2:c.1193T>A	I135: Brain
		NM_000179.2:c.1723G>T	I126: EC
*MUTYH*	P	NM_001128425.1:c.1187G>A	I085: Melanoma, myoxi malignant fibrous histiocytoma, CLL
			I127: CRC, non-endocrine PC
			I156: PS-A
		NM_001128425.1:c.536A>G	I148: Upper GI, URO (mediastinal germ cell tumour)
*NBN*	LP	NM_002485.4:c.156_157del	I176: BC, EC, NET-thyroid
*PALB2*	LP	NM_024675.3:c.2747_2748+4del	
	P	NM_024675.3:c.3113G>A	I129: Melanoma, respiratory, sarcoma (soft), RCC
			I134: NHL
*RAD51D*	LP	NM_002878.3:c.748del	I170: URO-seminoma, PC
*RET*	LP	NM_020975.4:c.2410G>A	I032: BC; respiratory, NET-thyroid (medullary); URO-urogenital carcinoma
*SDHA*	P	NM_004168.3:c.91C>T	I101: Brain

Somatic translocation identified in index case I079 is not included here. The structural variant caller DELLY was used to determine the breakpoints for complex (structural and copy-number variants)

More detailed presenting tumour-associated phenotype(s) is provided in Additional file 1: Table S1

*Abbreviations*: *ALL* acute lymphocytic leukaemia, *BC* breast cancer, *CLL* chronic lymphocytic leukaemia, *CRC* colorectal cancer, *EC* endometrial cancer, *GI* gastrointestinal, *HL* Hodgkin’s Lymphoma, *LP* likely pathogenic, *NET* neuroendocrine, *NHL* Non-Hodgkin’s Lymphoma, *OC* ovarian cancer, *P* pathogenic, *PC* prostate cancer, *PS-A* polyposis syndrome adenomatous or adenomas, *PS-H* polyposis syndrome hamartomatous or juvenile polyps, *RCC* renal cell carcinoma, *URO* urogenital

Most post-MDT meeting returned variants were missense (74.8%, 83/111). Large CNV or SV were less common with only eight reported following MDT meeting (Fig. 3C, D). This included a *RUNX1* translocation (index case I079) that was confirmed clinically as being of somatic origin. At least one variant was returned for 58 genes; variants within the *APC* (*n* = 7), *ATM* (*n* = 7), *MUTYH* (*n* = 6) and *MSH6* (*n* = 4) genes were the most frequently reported. As detailed in the methods, the virtual gene panel was revised during the course of the study (from panel A to panel B). Five of the returned variants were specific to genes present only within panel B all of which were classified as VUS. No post-MDT meeting reported variants were within genes specific to only panel A (Additional File 1: Table S4). We undertook a reanalysis of the 24 index cases, originally investigated with panel A, using panel B and did not identify any additional LP/P variants likely to explain the presenting phenotype. The only genes where multiple LP/P variants were identified were *ATM* (*n* = 2), *BRCA2* (*n* = 2), *DICER1* (*n* = 2), *MSH6* (*n* = 2), *PALB2* (*n* = 3) and *MUTYH* (*n* = 4) (Fig. 3, Table 3). Only two LP/P variants were returned to multiple index cases: NM_001128425.1(*MUTYH*):c.1187G>A (NP_001121897.1:p.Gly396Asp), also commonly referred to as G382D [35], observed in three (1.54%) cases; and NM_024675.3(*PALB2*):c.3113G>A (NP_078951.2:p.Trp1038Ter), known as a common pathogenic variant in the Australian population [36, 37], observed in two (1.03%) cases. Most index cases (68.5%, 63/92) with returned findings were diagnosed with multiple tumour types or tumour-associated phenotype(s). This trend was consistent when considering only those index cases with a returned LP/P variant (69%, 20/29) (Table 3, Additional File 1: Tables S1 and S4).

### Multidisciplinary team meeting review and changes to risk management

A MDT meeting review approach was used to clinically evaluate candidate variants prior to returning findings to the index cases. Approximately 25% (36/153) of variants presented for MDT meeting review reached consensus to exclude from the post-MDT research reports. Justification for excluding these variants were as follows: expert evaluation of the variant allele frequency in population databases and/or the literature; lack of gene-phenotype association; limited clinical utility of the variant; or preference for some FCCs to exclude VUS (especially VUS:C) from post-MDT meeting reporting. VUS that were considered clinically suspicious at the MDT meeting review, but did not a meet a LP/P classification were prioritised for additional work to clarify their clinical classification. These investigations led to the upgrade in clinical classification for variants in the *BAP1* and *MSH6* genes (Additional File 1: Table S4).

A LP/P germline finding led to changes in the risk management for 20 (10.3%) index cases and/or their families (Additional File 1: Table S8). This included results for two index cases identified at later stages of the study to carry the *PALB2* variant c.3113G>A known to be common in the Australian population and given the overwhelming evidence for disease causation of this variant in the Australian context. Clinical risk management changes that were initiated and/or recommended included altered surveillance strategies, prophylactic medication, risk-reducing surgery and/or cascade genetic testing. For nine (4.6%) index cases, the LP/P variant was considered as uninformative for the clinical context of the index case and their extended family. These variants were in genes associated with recessive conditions or a low (1.5 to threefold) increased risk for cancer, were already known to the index case, or of somatic origin as in index case I079.

### Health economics

A health economic analysis was performed to estimate and compare the annual costs and overall diagnostic yield of three hypothetical testing scenarios for affected individuals, defined as individuals presenting to an FCC on behalf of themselves, who will often (but not always) be the index case for the family (Fig. 1, Additional File 1: Table S5 to S7, inclusive). For the purposes of our study, all LP/P variants (both causal and secondary findings, excepting the somatic *RUNX1* translocation and two pathogenic variants that were already known to the participant) were considered clinically actionable with respect to cascade clinical testing becoming available for relevant family members. At a national level using State-based, aggregated, familial cancer testing data provided by Queensland Health and population data from the Australian Bureau of Statistics, we estimated that in 2020 there would have been 13,230 patients referred to an FCC, of whom 35.04% would have been offered germline genetic testing for suspected hereditary cancer. Scenario 1 (standard testing approach) was estimated to cost $4.9 M per annum and to be associated with an overall diagnostic yield of 3.5%; in the base case, this equates to 459 affected individuals nationally with an actionable variant detected (Additional File 1: Table S6). Scenario 2 (standard testing approach followed by WGS with an MDT meeting) was estimated to cost $45.3 M per annum with an overall diagnostic yield of 15.7% (2081 individuals nationally), and Scenario 3 (upfront WGS with an MDT meeting for complex cases and no MDT meeting for non-complex cases) was estimated to cost $43.9 M per annum with an overall diagnostic yield of 37.2% (4918 individuals nationally). Overall diagnostic yield was derived as the total number of cases identified in each scenario (green boxes in Fig. 1) divided by the initial number of affected individuals with suspected heritable cancer referred to an FCC (the first box in each scenario in Fig. 1). The cost per case with an actionable variant was estimated to be $10,712 for Scenario 1, $21,766 for Scenario 2 and $8,928 for Scenario 3 (Additional File 1: Table S6). It can be seen that Scenario 3 has a slightly lower annual cost than Scenario 2, and also that the number of actionable cases detected with Scenario 3 is more than twofold higher than with Scenario 2. The marginal cost per case with an actionable variant was significantly lower for Scenario 3 ($8,744) than for Scenario 2 ($24,894), when compared to Scenario 1 (Additional File 1: Table S7).

Sensitivity analyses applying different assumptions for key parameters in the economic analysis (Additional File 1: Table S6) found that the cost per actionable case detected in Scenario 3 was most sensitive to the rate of uptake of WGS and the diagnostic yield of WGS. The highest cost per actionable case detected for Scenario 3 ($19,083) was when the lowest plausible diagnostic yield for upfront WGS was applied (set to a value so that the overall diagnostic yield in Scenario 3 was equivalent to Scenario 2). The cost per actionable case detected for Scenario 2 was stable under the assumptions tested in the sensitivity analyses. Unsurprisingly, the estimates of total budget impact are most sensitive to the number of affected individuals referred to FCCs.

## Discussion

Our study showed that in an Australian clinical context, WGS delivers a clinically significant increase in the diagnostic yield of germline genetic testing for previously undiagnosed suspected familial cancer patients and their families. The increased diagnostic yield was attributable to identification of likely disease-causing LP/P variants in 10 index cases. These variants were identified in the following: non-coding regions not typically covered by conventional clinical genetic testing of the causative gene (two index cases); individuals where there were no recommended genetic tests for their clinical presentation (one index cases); and individuals where previous routine genetic testing had not provided a germline molecular genetic diagnosis (seven index cases). Whilst, the clinical presentation of the index case had some degree of association with the gene in which the causative variant was identified, the association was not enough to trigger testing for the gene in the index case. This highlights the imperfect relationship between a patient’s genotype and phenotype particularly for more heterogeneous cancer presentations. Similarly, whilst the non-coding (deep intronic) *BAP1* variant was identified in a family with a history of *BAP1*-related tumours, the second non-coding *APC* promoter variant was identified in an index case with a phenotype not consistent with the typical presentation, as previously reported. Specifically, germline variants that disrupt the *APC* 1B promoter are reported to be predominately associated with the gastric adenocarcinoma and proximal polyposis of the stomach (GAPPS) cancer predisposition syndrome rather than classical familial adenomatous polyposis as seen in this index case [38, 39].

Additional LP/P variants deemed as not causative for the initial presenting phenotype(s) were identified in 19 index cases. These included variants: in genes with a dominant tumour-associated phenotype triggering new risk management strategies (five index cases); providing a partial or incomplete genetic diagnosis (five index cases); and in genes with a predominantly recessive tumour-associated phenotype (nine index cases). It is also interesting to note that the majority (69%) of individuals identified to have a LP/P variants (causative and not causative of the condition), presented with multiple tumour-associated phenotypes.

During this study, we updated our virtual gene panel to reflect changes in clinical evidence. However, we conclude that the costs per case were not impacted by choice of panel. This is because reanalysis of cases with Panel B did not identify additional variants likely to explain the presenting phenotype, and all LP/P variants (explaining phenotype and secondary findings) that were incorporated into the economic analysis were identified in genes present in both panel A and panel B. The health economic analysis suggests that in Australia a national approach to testing based on upfront WGS with a virtual MDT for complex cases has a lower cost per actionable variant detected than either the standard testing approach, or an approach which triages patients to WGS after a standard testing approach has been applied. With an estimated total annual cost of $43.9 M, an upfront WGS approach to identifying heritable cancers would represent less than 0.5% of the total national annual expenditure on cancer diagnosis and treatment, estimated as $10.1 billion AUD in 2015–2016 [40]. In addition, although not captured in our analysis, it is expected that upfront WGS would be associated with a shorter time to genetic diagnosis for complex cases compared to the triaged approach to WGS. Our health economic analysis is limited by the fact that it was not feasible to undertake a cost-utility analysis based on the current study due to a highly heterogeneous cohort of rare cancers (and hence a wide range of downstream interventions and costs), and that no micro-costing or quality of life data were collected from the study participants. Nonetheless, the cost per actionable case detected for each scenario here is similar to values accepted by Australia’s National Health Technology Assessment committee responsible for assessing genetic and genomic testing in Australia [41]. Whilst a programmatic perspective has been taken to estimate the costs and diagnostic yield of the proposed model of care, the programme itself would rely on referral of affected individuals to FCCs and implementation of appropriate cancer risk management plans by relevant specialists.

Although not directly measured in our study, many of the critical contributions to delivering on this higher diagnostic yield may not have been achievable based solely upon the WGS approach itself and rather are due to the research-based context of our study. In particular, clinical evaluation of the index cases and the identified variants in an MDT meeting review setting proved a critical component of our study. These MDT meeting reviews provided an opportunity to discuss each case with a team of clinicians and researchers with diverse backgrounds, prompting: collection of additional biological samples to assist in germline investigations; suggestions of new therapeutic options or clinical testing; referral to research groups that could conduct functional assessment of variants or to enrol participants in other clinical trials; or identification of participants that would benefit most from future research endeavours. Whilst the benefits of incorporating research support with the MDT meetings is evident, it is important to note that the costs of these research activities were excluded from the economic and financial analyses because a programmatic perspective was taken.

In this study, we restricted our analysis to a virtual gene panel. However, an advantage of an upfront WGS-based approach is the relative ease at which the genomic data can be reanalysed, often at a reduced cost compared to initial testing, potentially leading to a further long-term increase in diagnostic yield [7, 8]. Previous comparative analyses have demonstrated that WGS of germline DNA (with sequence read depth) identifies essentially all variants detected by more targeted genetic sequencing, whilst providing additional findings [42–44]. These additional findings include variants within non-coding regions or more complex structural events such as novel gene fusions, large inversions, or promoter deletions. Improvements in diagnostic yield, ranging from 11 to 93% have been demonstrated via reanalysis with new (or revised) gene and variant level information; improved analytical techniques; or additional patient/familial phenotypic information [8]. The 26 index cases that met clinical criteria for a hereditary cancer syndrome in which a causative variant was not identified could be a key focus for such reanalyses. Generally, we expect that more disease-causing germline variants could be identified by expanding our analysis to regulatory regions or beyond the virtual panel to include other genes determined in the future to be relevant to hereditary cancer. Other targeted reanalyses could focus upon genes which undergo frequent updates within public databases, such as ClinVar [45],  or are notoriously challenging to analyse due to highly homologous pseudogenes (such as *PMS2*, *PTEN* and *STK11*) making alignment of sequence reads from these genes difficult and thus can mask the detection of disease-causing variants [46, 47]. Whilst WGS can be utilised to identify potential disease-causing variants in non-coding regions of the genome, it is important to note that the clinical interpretation of these variants is often difficult without some degree of prior characterisation, such as being located within a functionally demonstrated regulatory region or having been reported to co-segregate with disease [48, 49]. Likewise, the clinical interpretation of large complex genomic events can be limited by low resolution of genomic breakpoints. However, as bioinformatics methods improve and prioritisation strategies are developed, particularly for non-coding regions, the reanalysis of the existing WGS data for these individuals is likely to identify more candidate variants and help to clarify the classification of existing uncertain significance.

Future implementation of WGS into routine clinical care must consider several factors including technical challenges require to implement changes in workflow requirements for diagnostic testing laboratories, a matter that will extend beyond the diagnoses of hereditary cancer as well as establishing the triggers for data reanalysis or reinterpretation of clinical findings. Furthermore, there are a variety of ethical considerations for routine clinical WGS which include the long-term custodianship of genomic data, health and life insurance implications, maintaining ongoing consent for clinical or research purposes, and implications regarding the possible identification of secondary or incidental variants unrelated to the presenting phenotype of the tested individual. The latter issue can be limited through the use of virtual gene panels; however, it should be noted there is no currently adopted Australia-wide policy for the report of incidental or secondary findings by testing laboratories [50].

## Conclusions

In conclusion, using WGS we were able to increase the diagnostic yield of genetic testing for suspected familial cancer patients and their families in the Australian clinical setting. Economic analysis suggests that although total testing expenditure for upfront WGS would be nine times greater than current testing approaches, the cost per actionable case would be marginally lower. Any new model of care which includes WGS needs to be equitable, sustainable and cost-effective, to ensure the broadest access to care whilst maintaining diagnostic yield, and maintenance of a highly trained clinical and technical workforce. A coordinated national approach could allow the harmonisation of data, which has the potential to facilitate cascade testing across jurisdictions in a systematic and timely manner. Further economic analyses are required to determine the cost-effectiveness of upfront WGS for patients with suspected familial cancer in other countries considering implementing WGS for this purpose.

### Supplementary Information

**Additional file 1: Table S1.** Clinical information for all individuals recruited inclusive of index cases and family members. **Table S2.** Recorded risk-management strategies and related parameters. **Table S3.** Virtual gene panels used. **Table S4.** Variants identified within the 195 index cases that were prioritised for review at the multidisciplinary team meetings. **Table S5.** Estimation of average costs per affected individual for testing practice using targeted genes or gene panels, or whole genome sequencing. **Table S6.** Comparison of the costs and consequences of the different testing strategies. **Table S7.** Marginal analysis of the costs and consequences of the different testing strategies. **Table S8.** Summary of changes made to risk-management of index cases who were found to carry a likely pathogenic or pathogenic variant.

## Data Availability

Sequence data from individuals who have consented for further research has been deposited into European Genome-phenome Archive (EGA) repository under the study EGAS00001007045, https://ega-archive.org/studies/EGAS00001007045 [51].

## Abbreviations

AF: Allele frequency
B: Benign
CNV: Copy-number variant
FCC: Familial Cancer Centres
GAPPS: Gastric Adenocarcinoma and Proximal Polyposis of the Stomach
ICCon: Inherited Cancer Connect Partnership
IGV: Integrative Genome Viewer
Indel: Small insertions and deletions
LB: Likely benign
LP: Likely pathogenic
MDT: Multidisciplinary team
P: Pathogenic
SNV: Single-nucleotide variant
SV: Structural variant
VEP: Variant effect predictor
VUS: Variant of uncertain significance
WGS: Whole-genome sequencing
